# Warts

**Published:** 1901-03

**Authors:** 


### Abstracts and Selections.

### Warts.

Dr. G. M. Fitz calls attention to the excellent results which are obtained in the treatment of warts by solutions of chrysarobin. He first pares down the wart until there is rather profuse bleeding, and then applies a 10 per cent. solution of the drug, the vehicle being either ether or the ordinary gutta percha solution. As a matter of fact, chrysarobin is by no means the only effectual means of dealing with these often refractory conditions of epithelial hypertrophy. The essential part of the treatment is unquestionably the removal of the thickened epithelium. When this has been done the application of chromic acid or a collodion solution of salicylic acid will, in most cases, cause the disturbance of the growths within a few days.—*Exchange.*

